# Discontinuation of cART postpartum in a high prevalence district of South Africa in 2014

**DOI:** 10.1186/s13012-014-0139-3

**Published:** 2014-10-03

**Authors:** Lore Claessens, Anna Voce, Stephen Knight, Benn Sartorius, Ashraf Coovadia

**Affiliations:** Discipline of Public Health Medicine, School of Nursing and Public Health, College of Health Sciences, University of KwaZulu-Natal, Ground Floor, George Campbell Building, Durban, 4041 South Africa; Department of Paediatrics and Child Health, Faculty of Health Sciences, University of the Witwatersrand, 1 Jan Smuts Avenue, Braamfontein, Johannesburg, 2000 South Africa

**Keywords:** Prevention of mother-to-child transmission, cART, Discontinuation, Risk factors postpartum

## Abstract

**Background:**

Combination antiretroviral therapy (cART) is the current strategy to prevent mother-to-child transmission (PMTCT) of HIV. Women initiated on cART should continue taking treatment life-long or stop after cessation of breastfeeding depending on their CD4 cell count or on their World Health Organization (WHO) staging. Keeping people living with HIV on treatment is essential for the success of any antiretroviral therapy (ART) programme. There has been a rapid scale-up of cART in the PMTCT programme in South Africa. cART is supposed to be taken life-long or until cessation of breastfeeding, but premature or unmanaged discontinuation of cART *postpartum* is not unusual in South Africa and is confirmed by studies from around the world. Discontinuation of cART can lead to mother-to-child transmission (MTCT), drug resistance and poor maternal outcomes. The extent of this problem in the South African context however is unclear. This study aims to determine the prevalence of and identify risk factors associated with discontinuation of cART *postpartum* amongst women who were initiated on antiretroviral treatment during their index pregnancy.

**Methods:**

An observational analytic cross-sectional study design will be conducted in six health facilities in a high prevalence district in KwaZulu-Natal, South Africa over a period of 3 months in 2014. An interviewer-administered questionnaire will be used to collect data from mothers who initiated cART during their index pregnancy. The prevalence of discontinuation of cART *postpartum* will be measured, and the association between those who discontinue cART *postpartum* and independent variables will be estimated using multivariable-adjusted prevalence odds ratios for discontinuation.

**Electronic supplementary material:**

The online version of this article (doi:10.1186/s13012-014-0139-3) contains supplementary material, which is available to authorized users.

## Background

The national Prevention of Mother-to-Child Transmission (PMTCT) programme was implemented in South Africa in pilot sites in 2001 and rapidly expanded to the rest of the country in 2002 [1]. The PMTCT programme aims to reduce the vertical transmission of human immunodeficiency virus (HIV) and has four specific elements, namely 1) primary prevention of HIV in the mother, 2) prevention of unintended pregnancies among women living with HIV, 3) prevention of HIV transmission from mother to child, and 4) providing appropriate treatment and care for women living with HIV and their families [2]. Pregnant women living with HIV are identified during antenatal care (ANC) and if positive are initiated on a cART. Reducing the risk of vertical transmission during labour and delivery requires the recommended prophylaxis and adjustment of obstetric practices. *Postpartum*, the PMTCT programme aims to reduce vertical transmission by promoting breastfeeding with the administration of antiretroviral therapy to keep mothers and infants healthy through adequate follow-up visits for both mother and infant [2].

According to the latest national PMTCT guidelines, which were revised in March 2013, all pregnant women living with HIV are started on cART at the time of HIV diagnosis [2]. Women who have a CD4 count of ≤350 cells/mm^3^ or a WHO stage 3 or 4 of the disease are eligible for life-long cART (tenofovir disoproxil fumarate (TDF), emtricitabine (FTC)/3TC, EFV, ideally as fixed dose combination (FDC)) whilst women with a CD4 count of >350 cells/mm^3^ and a WHO stage 1 or 2 will stop cART 1 week after complete cessation of breastfeeding [2]. Women who were initiated on cART during pregnancy do not need any additional antiretroviral (ARV) medication during labour and delivery. The infant regimen comprises a single dose of nevirapine (NVP) at birth and which is continued for 6 weeks [2]. The daily dose of NVP is only continued beyond 6 weeks in those infants whose mothers are breastfeeding but who refused to take cART. NVP in these infants is continued for the duration of breastfeeding, unless the infant has been confirmed to be living with HIV [2].

Prior to March 2013, not all pregnant women living with HIV were initiated on cART. [3] Depending on their CD4 cell count and WHO staging, they were either initiated on life-long cART or on a dual ART regimen as prophylaxis. Although the emphasis was on cART being life-long, experience and informal reports suggest that discontinuation of cART *postpartum* is not unusual in South Africa. There is, however, limited evidence to verify the frequency of this practice in KwaZulu-Natal.

In April 2012, the WHO updated the ARV treatment policy to prevent vertical transmission of HIV in pregnant women. Three options were listed in this update: options A^a^, B^b^ and B+^c^. The revised South African PMTCT guidelines from 2013 are based on option B and not the alternative option B+. With option B+, all pregnant women living with HIV immediately start cART for life [4].

### Description of the problem

The selected health district in KwaZulu-Natal, South Africa has a population of 1 million [5] and an HIV prevalence of 39.8% (95% confidence interval (CI) 35.6%-44.3%) amongst pregnant women in 2011 as reported through the National Antenatal Sentinel HIV and Syphilis Prevalence Survey [6]. The evaluation of the effectiveness of the National PMTCT Programme on Infant HIV showed that at 6 weeks *postpartum* in KwaZulu-Natal, 29.4% (95% CI 25.5%-33.3%) of all pregnant women living with HIV were taking cART in 2010 [7]. Reported discontinuation of cART *postpartum* is not unusual and various factors have been identified as possible causes for this. These include but are not limited to the lack of time to process the diagnosis of HIV before initiation on cART, status disclosure, mental wellbeing, substance abuse and CD4 cell count. Retention on cART is one of the core indicators of the National Strategic Plan on HIV, STIs and TB, 2012-2016 [8]. The proportion of women who discontinue life-long cART in the *postpartum* period or before complete cessation of breastfeeding in this health district in KwaZulu-Natal is not known. Continuing cART for life or until breastfeeding has been completed leads to reduced mother-to-child HIV transmission, decreased drug resistance and improved maternal outcomes with less death, opportunistic infections and virologic failure [9],[10].

There is much evidence for the efficacy of cART; however, knowing the proportion of women who discontinue cART and the reasons for this in a real-life setting can improve the implementation of the revised policy. Now that increasingly more women are being initiated on cART, it is important to know what can be done to retain women in the ART programme even after delivery and the *postpartum* period. Gaining better insight of women's intentions regarding continuation of cART as well as their personal reasons and preferences can help inform further updates of the PMTCT guidelines and perhaps motivate policymakers to adopt the option B+ alternative in the future, should the evidence support this. Furthermore, the authors of a systematic review on unstructured treatment interruption of ART recommended that programmes should assess the causes and frequency of cART interruption as part of their routine monitoring [9].

### Research questions

The following research questions underpin the study:

1. What is the prevalence of discontinuation of combination antiretroviral therapy by 14 weeks *postpartum* in 2014 among women who were initiated on antiretroviral therapy during their pregnancy?
2. Which factors are associated with discontinuation of cART *postpartum*?

### Aim

The aim of this study is to determine the prevalence of and identify risk factors associated with discontinuation of cART by 14 weeks *postpartum* in women who were initiated on treatment during their index pregnancy and who attended immunisation clinics at one of the four selected primary health care facilities or two community health centres in KwaZulu-Natal, South Africa in 2014 to enable improvement of the quality of PMTCT-related care in the public health sector in KwaZulu-Natal.

### Specific objectives

The following are the specific objectives of the study:

1. To determine the prevalence of discontinuation of combined antiretroviral therapy by 14 weeks *postpartum* in women who were initiated on antiretroviral therapy during the index pregnancy
2. To determine the patient, condition, regimen, socio-economic and health care provider and system-related factors that are associated with discontinuation in comparison to the group of women who continued cART
3. To establish women's further intentions regarding continuation/discontinuation of cART *postpartum*
4. To identify preference regarding continuation/discontinuation of cART in women who are currently on prophylactic cART

### Conceptual framework

**Figure 1:**
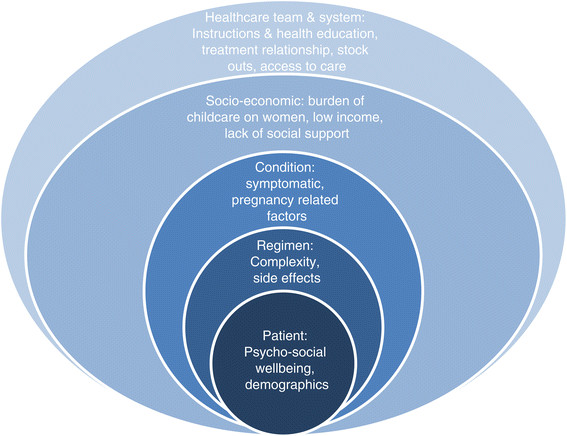
**Conceptual framework (adapted from the WHO framework for adherence to long-term therapies applied to HIV and AIDS) [**[11]**].**

Although this study looks at discontinuation and not at adherence, it is mainly based on a conceptual framework developed by the WHO to study and improve adherence to long-term HIV/AIDS therapies. According to this framework, there are four main groups of predictors to adherence 1) regimen, 2) patient, 3) provider-patient relationship and 4) system-of-care-related factors [11]. Based on these groups, five categories were derived and the following framework was developed (Figure 1). It is assumed that factors that need to be addressed when studying or improving adherence can also be applied in the study of discontinuation as the two are inter-related. The exposure variables measured in this study are based on this framework. A conceptual framework permits the measurement of the association not only between individual factors and the outcome but also between categories and outcome.

## Methods

### Study setting

This study will be conducted at six facilities of which four are primary health care clinics and two are community health centres (CHCs). The two CHCs are the two largest primary health care sites in the urban part of the selected district in KwaZulu-Natal. These six facilities are situated in a variety of areas from rural to semi-rural and urban. All six facilities offer antenatal care including PMTCT, postnatal care and child health care including immunisations. Included in the PMTCT services is initiation on cART for all pregnant women. Based on the District Health Information System (DHIS) data, these facilities provide most of the immunisation services to the infants in the selected district. All facilities are located in a high HIV prevalence district which is situated on a major transport route connecting two of South Africa's biggest metros, Durban and Johannesburg.

### Study design

**Figure 2:**
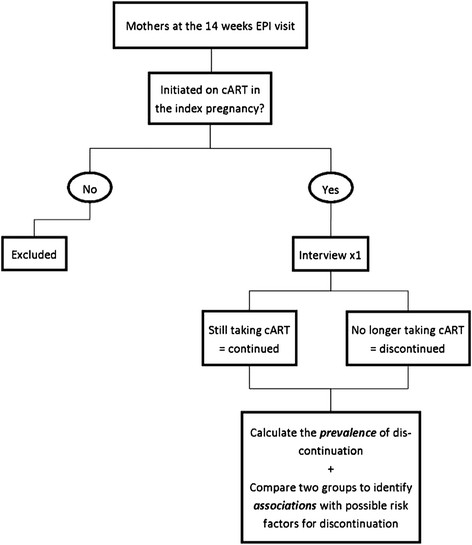
**Schematic diagram of the proposed cross-sectional study.**

An observational, analytic cross-sectional study design will be used as presented in Figure 2.

### Target population

The target population to which the results of this study could be generalised is women of reproductive age, defined as 15-49 years old, living with HIV, who are pregnant and who are eligible for cART initiation in KwaZulu-Natal according to national ART guidelines.

### Study sample

A consecutive sampling method will be used in recruiting the sample. From all non-probability sampling methods, this is the best method as it seeks to include all accessible subjects, which makes the sample more representative of the entire population. All women who fit the inclusion criteria at the time of data collection will be invited to participate in the study.

### Sample size

To detect a hypothesised 20% difference (translates into a prevalence rate ratio of 1.4 or a prevalence odds ratio of 2.3) in prevalence between those who discontinued versus those who continued with cART (two independent proportions test) with 80% power (1-β [type 2 error probability]) and 95% confidence interval (or 5% α error probability [type 1]), assuming a discontinuation proportion of 50% in the unexposed group (yields the largest possible sample size if previous estimates are not available to suggest an estimate substantially different from 50%), yields a total required sample size of 186. Assuming a non-response rate of 20% (assuming random non-response), the final total sample size required would be 224 subjects.

### List of variables

The outcome variable is the discontinuation of cART. Table 1 presents the independent variables per category based on the conceptual framework. Associations between discontinuation of cART and the independent variables will be measured in order to identify risk factors for the discontinuation of cART *postpartum*.

**Table 1:** **List of variables per category**

Category	Variables
Patient	Age
	Gravidity
	Parity
	Number of children alive
	Alcohol and drug abuse
	Psychological wellbeing/feeling happy
Regimen	Regimen
	Side effects
	Switch in regimen
	Time between HIV diagnosis and cART initiation
	Duration of cART in pregnancy
	Time of discontinuation
Condition	CD4 cell count at initiation
	TB treatment
	Multiple pregnancy
	Mode of delivery
	Pregnancy complications
	Pregnancy-induced hypertension/pre-eclampsia/eclampsia
	Preterm labour/post-term labour
	Gestational diabetes
	Stillbirth
	Intra-uterine growth retardation/low birth weight
	Haemorrhage (ante- or *postpartum*)
	Others
	Own health
Socio-economic	Marital status
	Employment status
	Access to income
	Highest level of education
	Treatment buddy
	Disclosure of status
	Health of possible previous children
	Health of the newborn
	HIV status of the newborn
	Infant feeding method
Health care worker and system	Health education
	Stock outs
	Relationship health care provider
	Planned follow-up
	Travel costs to CHC
Qualitative variables	Reason for discontinuation
	Intention for future
	Preference regarding continuation/discontinuation
	Advise to promote continuation

### Data collection and analysis

The six research assistants who have been employed to conduct the data collection through interviewer-administered questionnaires are currently conducting interviews in all six facilities simultaneously. Women who attend the 14-week immunisation visit with their infants and who have been initiated on cART in their index pregnancy are invited to participate in the study.

Data processing and analysis will be conducted using Stata 13.0 [StataCorp. Stata Statistical Software: Release 13. College Station, TX, USA]. Both descriptive and analytic statistics will be employed. The variables will be summarised by using appropriate graphs and frequency distribution tables. The prevalence of discontinuation of cART will be estimated along with 95% confidence intervals. Differences in the mean values of continuous explanatory variables by discontinuation status will be assessed using Student's *t*-test. If the data are not-normal than the non-parametric Wilcoxon rank-sum test will be used instead. Pearson's chi-square (*χ*^2^) test will be used to identify significant association between categorical variables and the outcome. A *p* value <0.05 will be considered as statistically significant. Association between each explanatory variable and the outcome will be tested using a bivariate logistic regression. Variables significantly associated with the outcome at 15% level (*p* < 0.15) will be included in the final multivariable model. Potential confounders will be included in the final model. Co-linearity between covariates will be assessed using variance inflation factors (VIF). Model fit and diagnostics will also be assessed to ensure model adequacy. The final logistic regression model will also be evaluated in terms of its predictive power for discontinuation of cART. Coefficients will be exponentiated to present prevalence odds ratios and 95% confidence intervals.

### Limitations

**Figure 3:**
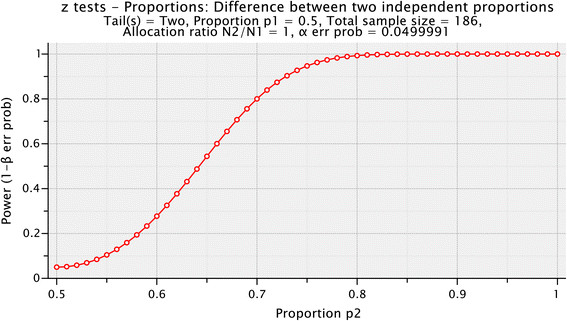
**Power for the difference between two independent proportions.**

The study will not be powered to detect any difference between the two groups below 20% with 80% power. The rapid reduction in power when the difference drops below 20% (i.e. *p*_[exposed]_ < 0.7 versus *p*_[unexposed]_ = 0.5) can be seen in Figure 3.

### Study status

Ethics approval from the University of KwaZulu-Natal Biomedical Research Ethics Committee and the KwaZulu-Natal Provincial Department of Health has been obtained. Data collection started in June 2014 and will continue until the end of August 2014 at which point the achieved sample size will be evaluated and a decision made on whether the data collection period will be extended or not.

## Endnotes

^a^Treatment or prophylaxis dependent on CD4 count: CD4 ≤ 350 cells/mm^3^ or WHO stage 3 or 4 regardless of CD4 count, life-long ART. CD4 > 350 cells/mm^3^ and WHO stage 1 and 2: antenatal and intra-partum prophylaxis (AZT, sdNVP, TDF/FTC). Extended infant NVP syrup for breastfed infants [4].

^b^All HIV-infected pregnant women initiated on ART regardless of CD4 count: CD4 ≤ 350 cells/mm^3^ or WHO stage 3 or 4, life-long ART. CD4 > 350 cells/mm^3^ and WHO stages 1 and 2, stop ART after delivery if formula feeding or after cessation of breastfeeding [4].

^c^Life-long ART for all HIV-infected pregnant women regardless of CD4 count [4].

## Authors' contributions

LC is the main author who developed the study proposal including literature review, managed all the ethics approval applications and recruited and managed the research assistants. AV, AC and SK reviewed and participated in the development of the proposal and assisted in the design of the study. BS assisted in the design of the study and carried out the sample size calculations. All authors read and approved the final manuscript.

## Authors’ original submitted files for images

Below are the links to the authors’ original submitted files for images. Authors’ original file for figure 1 Authors’ original file for figure 2 Authors’ original file for figure 3

## Abbreviations

3TC: lamivudine
AIDS: acquired immunodeficiency syndrome
ANC: antenatal care
ARV: antiretroviral
cART: combination antiretroviral therapy
CI: confidence interval
DHIS: District Health Information System
EFV: efavirenz
FDC: fixed dose combination
FTC: emtricitabine
HIV: human immunodeficiency virus
NVP: nevirapine
PMTCT: prevention of mother-to-child transmission of HIV
TB: tuberculosis
TDF: tenofovir disoproxil fumarate
WHO: World Health Organization
