# Supplementary figures and images for: Discontinuation of cART postpartum in a high prevalence district of South Africa in 2014
Source: Implement Sci. 2014 Oct 3;9:139. doi: 10.1186/s13012-014-0139-3 (PMC4198759; doi:10.1186/s13012-014-0139-3)

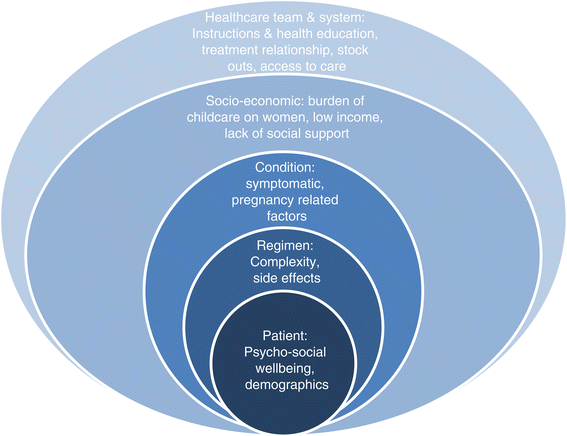

Supplement: Supplementary file 1 — Authors’ original file for figure 1 [file 13012_2014_139_MOESM1_ESM.gif]

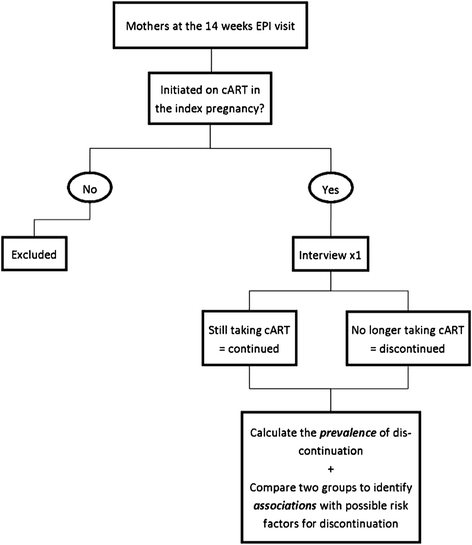

Supplement: Supplementary file 2 — Authors’ original file for figure 2 [file 13012_2014_139_MOESM2_ESM.gif]

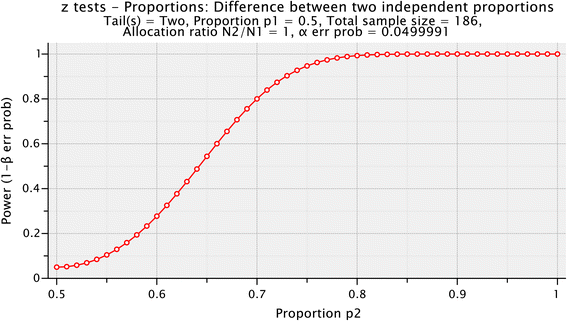

Supplement: Supplementary file 3 — Authors’ original file for figure 3 [file 13012_2014_139_MOESM3_ESM.gif]
